# Management of Antithrombotic Agents in Oral Surgery

**DOI:** 10.3390/dj3040093

**Published:** 2015-10-06

**Authors:** Maria Martinez, Dimitrios A. Tsakiris

**Affiliations:** Diagnostic Hematology, University Hospital Basel, CH-4031 Basel, Switzerland; E-Mail: maria.martinez@usb.ch

**Keywords:** oral anticoagulants, dental surgery, oral surgery, DOAC, direct oral anticoagulants, NOAC, new oral anticoagulants, heparins, antiplatelet agents

## Abstract

Systemic anticoagulation with intravenous or oral anticoagulants and antiplatelet agents is an efficient treatment against thromboembolic or cardiovascular disease. Invasive dental procedures or oral surgery might be associated with bleeding complications if carried out under anticoagulants. Patients on vitamin K antagonists, new direct anticoagulants or antiplatelet agents having dental interventions with low-risk for bleeding do not need interruption of anticoagulation. In case of bleeding complications local hemostatic measures, such as local surgical sutures, fibrin glue, local antifibrinolytic treatment with tranexamic acid, or e-aminocaproic acid suffice to stop bleeding. In patients with high risk of bleeding an individual assessment of the benefit/risk ratio of interrupting anticoagulation should be carried out. Bridging the long-term anticoagulation with short-term anticoagulants should be planned according to national or international guidelines. The introduction of the newer direct oral anticoagulants having more flexible pharmacokinetic properties has facilitated bridging, allowing short-term interruption without increasing the risk of relapsing thrombotic or cardiovascular events.

## 1. Introduction

Several medical conditions, such as venous thromboembolism or cardiovascular events, need antithrombotic drugs as prophylaxis or treatment against relapse. For many decades, systemic anticoagulation was applied by using vitamin K antagonists (VKA) and heparins on one side and aspirin as an antiplatelet agent on the other. New direct oral anticoagulants (DOAC), targeting single coagulation enzymes, such as thrombin or factor Xa have been developed in the last ten years, being as good as VKA in preventing thromboembolic events and carrying a lower risk for major bleeding complications (Table 1). Managing of anticoagulation before and after surgical or invasive diagnostic procedures is always a challenge, requiring a bridging strategy, which would allow surgery without bleeding complications and keep, in parallel, the risk of thrombosis at the lowest level. Major surgery in this context has been extensively investigated in the literature. Oral surgery is lacking large studies, whereas increasing numbers of scientific reports with small cohorts, randomized or observational studies have appeared in the last few years. The management of patients receiving VKA and requiring dental invasive procedures is quite well documented in the literature [1]. The risk of bleeding is usually minor and self-limiting, and does not outweigh the risk of thromboembolic events. Evidence is not that clear, though, for patients treated with DOAC.

The development of DOAC focused on eliminating some complications and disadvantages associated with the older anticoagulants. The new drugs have a wider therapeutic range, can be applied at fixed doses, act independently of age, sex, and body weight, and do not need technical monitoring, even if in some cases assaying of the anticoagulant effect may be helpful. Direct specific antidotes have also been developed and studied in phase III studies, but are not yet available on the market [2].

**Table 1:** The new direct oral anticoagulants (DOAC) and their mode of action.

	Apixaban (Eliquis®)	Rivaroxaban (Xarelto®)	Dabigatran (Pradaxa®)	Edoxaban (Lixiana®)	Betrixaban
**Action**	Anti-FXa	Anti-FXa	Anti-FIIa	Anti-FXa	Anti-FXa
**Cmax**	3–4 h	2–3 h	2 h	2 h	3 h
**T ½**	8–15 h	7–11 h	8–15 h	10 h	20 h
**Elimination**	27% renal 73% hepatic	33% renal active 33% hepatic 33% renal inactive	80% rena l20% hepatic	35% renal 65% hepatic	5% renal 95% hepatic
**Dosing**	bid	qd	qd, bid	qd	qd
**Monitoring**	no	no	no	no	no
**Interaction**	CYP3A4, P-gp	CYP3A4, P-gp	P-gp	P-gp	CYP3A4
**Antidote**	Andexanet alfa	Andexanet alfa	Idarucizumab	Andexanet alfa	Andexanet alfa

## 2. Intravenous and Oral Anticoagulants

### 2.1. Oral Vitamin-K-Antagonists (VKA)

The discovery of warfarin (Coumadin^®^) [3] began, almost by chance, in the early 20th century in Canada by examining the reason why healthy cattle died from internal bleeding. The reason was the feeding of the animals with moldy sweet clover hay and a substance therein which acted as a Vitamin-K-Antagonist. They inhibit the posttranslational carboxylation of glutamic acid residues of coagulation factors II, VII, IX and X and are acting thus upstream from thrombin in the coagulation cascade. Warfarin has a long plasma half-life of 40 h and an even a longer anticoagulation effect. Additional VKA available in Europe are phenprocoumon (Marcoumar^®^), acenocoumarol (Sintrom^®^) and phenindione with half-lives of 160 h, 16 h and 7 h, respectively. All of them depend on multiple hepatic enzyme-systems for their metabolism and thus interact with many co-medications, which can complicate the control of the anticoagulation effect. In addition gastrointestinal absorption of vitamin K is depending on many factors, such as diet or gastrointestinal disease.

#### 2.1.1. VKA and Oral Surgery

Anatomically, the mouth region, receiving a four-fold blood patency with a mucosa which can be easily injured, is prone to easy bruising and bleeding. Saliva containing lysozymes and other proteolytic enzymes can assist fibrinolysis and perpetuate bleeding. On the other hand, saliva expresses tissue-factor-like substances, which facilitate thrombin generation, thus adding to the hemostatic effect. Addition of anticoagulants might disturb this balance and cause prolonged bleeding in cases of dental surgery. Several clinical studies have investigated the bleeding risk of patients with oral surgery while being under anticoagulation with VKA [1,4,5,6,7]. In a recent prospective study [8], about 100 patients on Vitamin-K-Antagonists were not discontinued and approximately another 100 patients were bridged with low molecular weight heparin and reached, the day of procedure, an INR < 1.5. Treatment with LMWH was discontinued at least 12 h before intervention. Only a few post-interventional minor bleeding events occurred within the two groups. Even if the group with permanent anticoagulation had a few more bleedings, there was no significant difference between the two groups. All minor bleeding complications were easily resolved with local hemostatic measures. Some other studies showed very similar results [4,9,10]. Official guidelines do not suggest interruption of VKA for low risk dental procedures, local hemostatic measures, such as surgical hemostasis, suturing, fibrin glue, local antifibrinolytics with application of tranexamic acid, or e-aminocaproic acid as mouth rinses, would be sufficient to secure hemostasis [1]. In any case, though, no anticoagulation is safer than operating under anticoagulants. For short-term anticoagulation periods, whenever possible, invasive dental procedures should be postponed until after the end of the anticoagulation period.

### 2.2. Heparins

After the discovery of heparin [3] in the early 20th century, this drug was widely used for anticoagulant therapy since the 1950s. Unfractionated Heparin (UFH) is given intravenously and low molecular weight heparin (LMWH) is usually administered subcutaneously. Both have a rapid onset of anticoagulation, need a cofactor, antithrombin, and after binding to it they can exert the anticoagulation effect mainly on thrombin, but also on other coagulation factors. LMWH exerts a stronger anti-Xa-effect than UFH. Some advantages of LMWH compared to UFH are the longer half time, better bioavailability, resulting in more predictable dose response, and the subcutaneous application with the possibility to apply it in outpatients once or twice daily without the need for hospitalization in cases of bridging. UFH need regular laboratory monitoring for daily dosing, LMWH do not require monitoring except in cases of potential accumulation or heparin resistance.

#### 2.2.1. Heparins and Oral Surgery

A randomized study [8] and a large meta-analysis [11] showed that patients treated with LMWH in low-dose regimes for prophylaxis did not have significantly more bleeding complications than those treated with VKA. Dosing LMWH or UFH at a therapeutic intensity is expected, though, to cause increased bleeding. Since no direct data on this treatment are available for patients undergoing dental procedures, this is extrapolated from the experience on other kinds of minor or major surgery. Management in these cases should be chosen individually. In general, UFH can be stopped for some hours without increasing the risk for thrombosis. LMWH dosing schedule can be chosen in a way that the invasive procedure is carried out at the time of lowest level of LMWH activity, thus ensuring adequate hemostatic function. 

### 2.3. Newer Direct Oral Anticoagulants (DOAC) 

In contrast to the Vitamin-K-antagonists, the direct oral anticoagulants target specific single enzymes in the coagulation cascade. There were several studies that showed that DOAC were as effective as the oral VKA in preventing thrombotic events with fewer major or fatal bleeding complications [12,13,14,15,16]. 

Factor-Xa-inhibitors (rivaroxaban, apixaban, edoxaban) are small molecules that bind the part of FXa that catalyzes prothrombin to thrombin, therefore inhibiting thrombin generation. They are able to inhibit the free and bound FXa to the clot. Thus, they are acting selectively and reversibly. All three FXa inhibitors launched as antithrombotics reach the maximum concentration about 3 h after ingestion. They have a short elimination half-life (9–13 h), which, in elderly people or in patients with renal impairment, can be prolonged.

Dabigatran [17] is a selective competitive direct thrombin inhibitor, which binds free, fibrin-bound and clot-bound thrombin. The peak time is reached at 2 hours after ingestion. Approximately 80% of the drug is excreted in urine with an elimination half-life of 12–17 h, so that patients with impaired renal function may have longer elimination half-lives.

Specific antidotes have already been produced for all DOAC (Table 1) and have been tested in phase III clinical studies with favorable results and are awaiting market-launch [2].

#### 2.3.1. DOAC and Oral Surgery

In a secondary analysis of the RE-LY study (dabigatran in atrial fibrillation) [12], 10% of the patients who needed an invasive procedure needed dental intervention; there were no significant differences in the bleeding rates between the Dabigatran and the warfarin group, however, the intervention was performed after cessation of Dabigatran for at least 24 h. In another registry [18], the peri-interventional management of the new oral anticoagulants in daily care was analyzed. Of 2170 registered patients, 116 patients had dental interventions (extractions, implants) and other 25 had dental interventions other than extraction. No major bleeding complications occurred within the group of patients, which did not interrupt DOAC. These data suggest that minor procedures with a low risk of bleeding, such as dental procedures, in which warfarin can be safely continued, that DOAC can be continued as well, even if others recommend a cessation of the drug 24 h before intervention with a low bleeding risk [19,20,21,22]. Patients with risk of drug accumulation, such as those with renal insufficiency or additional medication and interaction with DOAC, need more attention. In these cases, preoperative cessation of the drug for more than 24 h might be necessary. All manufacturers have published recommendations in their investigator’s brochure for precautionary measures in surgical procedures with low or high risk of bleeding. The procedure should preferably be planned at the time of lowest level of plasma activity of the DOAC and should not be performed at the peak level. For patients undergoing oral/maxillofacial surgery, where the bleeding risk is significant, DOAC should be discontinued for at least 24 h. In general, the predictable pharmacokinetic profiles and the relative short half-lives of DOAC make transitional anticoagulation for the peri-interventional period quite easy. No bridging with other anticoagulants is normally needed, e.g., changing DOAC to LMWH does not add any benefits and is unnecessary.

## 3. Antiplatelet Agents

Aspirin (acetylsalicylic acid) was the first drug to inhibit platelet function and is still the most prescribed. In low and high doses, it irreversibly blocks cyclooxygenase 1 (COX-1) in the platelets and, consequently, inhibits the formation of thromboxane. Later on, the thienopyridines, as P2Y12 receptor antagonists of the platelet surface, were developed and came on the market. Ticlopidine was the first one registered, but due to side effects it was quickly replaced by clopidogrel. Both are prodrugs and need to be hydrolyzed first and metabolized in the liver to produce the active metabolites. This resulted in a considerable individual variability concerning the antiplatelet effect. Looking for drugs with faster onset and less inter-individual variability, ticagrelor and prasugrel were developed with no or only partial transformation needed until the active substance becomes available after absorption. Biological resistance to ticagrelor or prasugrel is practically unknown. They protect more efficiently than clopidogrel from cardiovascular events, but at the cost of safety, with increased rates of bleeding complications [23]. 

### 3.1. Oral Surgery and Antiplatelet Agents

There is a considerable amount of scientific evidence showing that simple dental interventions can be safely performed under ongoing antiplatelet treatment [24]. Depending on the indication of aspirin prophylaxis (secondary or primary), the risk to suffer a thrombotic event after stopping antiplatelet treatment might be high and has to be evaluated against the possible bleeding risk [25,26,27,28]. Patients with complicated catheter interventions are at high risk for relapsing cardiovascular events and have to be put on dual antiplatelet treatment for short or longer term (e.g., after drug eluting stenting). In these patients, interrupting one of the antiplatelet agents is related to a high risk of in-stent thrombosis [29].

In a prospective study [25], which analyzed the bleeding complications after tooth extraction under antithrombotic treatment with aspirin or clopidogrel alone or under dual antiplatelet treatment, compared to a control group with no antiplatelet agents, the bleeding risk was higher in the group with dual antiplatelet treatment compared with no treatment or with a single antiplatelet drug. A main cause for immediate or late post-interventional bleeding was periodontitis with related tissue fragility and hyperemia. In particular, all patients on dual antiplatelet therapy with periodontitis developed prolonged post-extraction bleeding. All bleeding complications were successfully treated with local hemostatic measures. This study shows that, for low-risk dental extraction, there is no need to interrupt antiplatelet treatment and that it is possible to estimate the benefit/risk ratio in patients with higher individual bleeding risk. More elaborate local hemostatic measures (e.g., suturing of the wound, application of hemostatic gauzes, local use of tranexamic acid) are efficient in such cases, thus avoiding interruption of the antiplatelet treatment. Alternatively, periodontitis might be treated in advance, if time permits, to correct for the increased bleeding risk due to inflammation and hyperemia. 

## 4. Conclusions

- Patients on anticoagulants in the form of vitamin K antagonists, new direct anticoagulants, or antiplatelet agents, having a low risk dental intervention, do not need interruption of anticoagulation, as they have a low risk of bleeding (Table 2). In case of bleeding complications, local hemostatic measures, such as local surgical sutures, fibrin glue, local antifibrinolytic treatment with tranexamic acid, or e-aminocaproic acid suffice to stop bleeding. For short-term anticoagulation periods, dental surgery should be, preferably, postponed until the end of anticoagulation whenever possible.
- In patients needing oral surgery or dental interventions with a high risk of bleeding, an individual assessment of the benefit/risk ratio of interrupting anticoagulation should be carried out. Bridging the long-term anticoagulation with short-term anticoagulants should be planned according to national or international guidelines.
- The introduction of the newer direct oral anticoagulants, having more flexible and reproducible pharmacokinetic properties, has facilitated bridging, allowing short-term interruption without increasing the risk of relapsing thrombotic or cardiovascular events.
- Patients on single antiplatelet agents do not need to interrupt their treatment before dental procedures with low bleeding risk. In cases of dual antiplatelet treatment or in invasive dental procedures with a high bleeding risk, an individual assessment of the benefit/risk ratio of interrupting anticoagulation should be carried out.

**Table 2:** Dental procedures classified according to their bleeding risk*.

Low bleeding risk	High bleeding risk
Scaling or root canal planing Endodontic treatment Dental restorative treatment Simple extraction Surgical procedures less than 45min	Surgical tooth extraction Multiple tooth extractions Complex oral surgery Head and neck surgery

*Adapted from *Thean et al. Aust Dent J 2015 *[30].
